# Effect of COVID-19-restrictive measures on ambient particulate matter pollution in Yangon, Myanmar

**DOI:** 10.1186/s12199-021-01014-3

**Published:** 2021-09-18

**Authors:** Win-Yu Aung, Paw-Min-Thein-Oo, Zaw-Lin Thein, Sadao Matsuzawa, Takehiro Suzuki, Yo Ishigaki, Akihiro Fushimi, Ohn Mar, Daisuke Nakajima, Tin-Tin Win-Shwe

**Affiliations:** 1 Department of Physiology, University of Medicine 1, Kamayut Township, 11014 Yangon, Myanmar; 2 Health and Environmental Risk Division, National Institute for Environmental Studies, 16-2 Onogawa, Tsukuba, Ibaraki, 305-8506 Japan; 3 Graduate School of Informatics and Engineering, The University of Electro-communication, Chofu, Tokyo, 182-8585 Japan

**Keywords:** Air quality, COVID-19, Mobile PM Sensor, Stay-At-Home, Telework, Yangon

## Abstract

**Background:**

Particulate matter (PM) is recognized as the most harmful air pollutant to the human health. The Yangon city indeed suffers much from PM-related air pollution. Recent research has interestingly been focused on the novel subject of changes in the air quality associated with the restrictive measures in place during the current coronavirus disease-2019 (COVID-19) pandemic. The first case of COVID-19 in Myanmar was diagnosed on March 23, 2020. In this article, we report on our attempt to evaluate any effects of the COVID-19-restrictive measures on the ambient PM pollution in Yangon.

**Methods:**

We measured the PM concentrations every second for 1 week on four occasions at three study sites with different characteristics; the first occasion was before the start of the COVID-19 pandemic and the remaining three occasions were while the COVID-19-restrictive measures were in place, including Stay-At-Home and Work-From-Home orders. The Pocket PM_2.5_ Sensor [PRO] designed by the National Institute for Environmental Studies (NIES), Japan, in cooperation with Yaguchi Electric Co., Ltd., (Miyagi, Japan) was used for the measurement of the ambient PM_2.5_ and PM_10_ concentrations.

**Results:**

The results showed that there was a significant reduction (*P* < 0.001) in both the PM_2.5_ and PM_10_ concentrations while the COVID-19-restrictive measures were in place as compared to the measured values prior to the pandemic. The city experienced a profound improvement in the PM-related air quality from the “unhealthy” category prior to the onset of the COVID-19 pandemic to the “good” category during the pandemic, when the restrictive measures were in place. The percent changes in the PM concentrations varied among the three study sites, with the highest percent reduction noted in a semi-commercial crowded area (84.8% for PM_2.5_; 88.6% for PM_10_) and the lowest percent reduction noted in a residential quiet area (15.6% for PM_2.5_; 12.0% for PM_10_); the percent reductions also varied among the different occasions during the COVID-19 pandemic that the measurements were made.

**Conclusions:**

We concluded that the restrictive measures which were in effect to combat the COVID-19 pandemic had a positive impact on the ambient PM concentrations. The changes in the PM concentrations are considered to be largely attributable to reduction in anthropogenic emissions as a result of the restrictive measures, although seasonal influences could also have contributed in part. Thus, frequent, once- or twice-weekly Stay-At-Home or Telework campaigns, may be feasible measures to reduce PM-related air pollution. When devising such an action plan, it would be essential to raise the awareness of public about the health risks associated with air pollution and create a social environment in which Telework can be carried out, in order to ensure active compliance by the citizens.

## Background

Air pollution is the greatest environmental risk to human health and represents the world’s fourth leading cause of premature deaths [1]. It has become a global public health emergency that affects people of all ages in every part of the world [2]. An increasing range of adverse health effects has been linked to air pollution, even low levels of pollution, and this is especially true of airborne particulate matter (PM). The World Health Organization (WHO) estimates that about seven million people die each year from excessive exposure to ambient PM [3]. Currently, among the six major air pollutants, PM is considered to be the most hazardous to human health [4].

Long-term exposure to PM pollution has been reported to be potentially associated with an increase in cardiopulmonary mortality [5], impairment in cognitive functions [6], diabetes mellitus [7], and adverse birth outcomes [8]. Recently, PM_2.5_ was found to be related to in vitro toxic potentials, such as oxidative potential, inflammatory response, aryl hydrocarbon receptor agonist activity, and deoxyribonucleic acid damage [9].

On the other hand, the year 2020 has proven to be one of the most catastrophic for the global population because of the coronavirus disease-2019 (COVID-19) pandemic, which is considered as the greatest challenge that humans have ever faced since World War II [10]. Severe acute respiratory syndrome coronavirus 2 (SARS-CoV-2) has been identified as being responsible for the outbreak of COVID-19. On December 31, 2019, the Chinese authorities notified the WHO of several cases of an unusual type of pneumonia in Wuhan City [11]. On January 30, the WHO declared a worldwide public health emergency of international concern and on March 1, the WHO declared the COVID-19 outbreak as a global pandemic [11].

While the outbreak was confirmed in mid-January in the Southeast Asian region, the first case of COVID-19 in Myanmar was confirmed on March 23, 2020 [12]. During the month of March, people were advised to avoid mass gatherings and campaigns for promoting personal hygiene measures such as frequent hand washing and social distancing were introduced and encouraged nationwide. On March 29, visits by people from all other countries were restricted by temporary suspension of issuance of all types of visas, except visas for diplomats accredited to Myanmar, United Nations official residents in Myanmar, and crew of ship and aircraft operations to and from Myanmar. From April 18, 2020, the Ministry of Health and Sports (MOHS) enforced some further restrictive measures, such as Stay-At-Home and Work-From-Home orders; closure of factories, universities, restaurants, and shops; avoidance of social gathering of more than 5 people; and night curfew orders from midnight to 4 am [12].

These measures during the COVID-19 pandemic brought about an unprecedented effect on the global air quality, and changes in the air quality related to COVID-19-restrictive measures became a novel and interesting research topic. A thorough review of the literature on the effects of COVID-19-restrictive measures on the air quality revealed three striking points; (1) the degrees of changes of the PM concentrations were variable, ranging from noticeable reduction [13–17] or a slight reduction to even an unexpected increase [18–21], as compared to the PM concentrations recorded before the start of the COVID-19 pandemic; (2) the changes in the PM concentrations during different measurement periods during the COVID-19 pandemic could be inconsistent [18, 22]; and (3) even within the same nation with uniform restrictive measures in place, there could be unpredictable changes in the PM concentrations in different locations with different characteristics [22].

While there are a number of reports regarding the effects of COVID-19-restrictive measures on airborne PM pollution, we considered it necessary to conduct a local study for our own country. Accordingly, we chose Yangon city, a metropolitan city that is the most densely populated part of the metropolitan area of Yangon Region, and also was the epicenter of the COVID-19 outbreak. We previously assessed the ambient PM_2.5_ and PM_10_ concentrations in seven townships of the city and recorded levels higher than the limits stipulated by the WHO guideline [23, 24]. Recently, according to one report, Myanmar showed a 28.6% reduction of the PM_2.5_ concentration during an initial short period of community lockdown (March 27 to April 30, 2020) as compared to the value recorded in the corresponding period in the previous year, 2019 [25].

Based on the aforementioned three salient points, we randomly selected three study sites with different characteristics in Yangon city. We verified the changes in the ambient PM concentrations during three periods of 2020 in which COVID-19-restrictive measures were in place, as compared to the values recorded prior to the onset of the COVID-19 pandemic, and also as compared to the values recorded during corresponding periods of the year in the previous year 2019, and to compare the percent changes among the three different selected sites. We expected that the findings of our study would provide useful information for judging the effectiveness of restrictive measures in reducing airborne PM pollution in the metropolitan area of Yangon.

## Material and methods

### Study area

For our present study, we selected Yangon city to evaluate the impact of COVID-19-restrictive measures on the ambient PM pollution in a metropolitan city of Myanmar. The city is a commercial and industrial center, and the most heavily populated in the country. It covers a surface area of nearly 600 kilometer squared, and is home to over 54 million people, i.e., 10% of the total population of the country [26]. It is situated at a latitude of 17.1°N and longitude of 96.1°E, in the southern part of Myanmar, bordered by the Bago Region to the North and East, the Gulf of Martaban to the South, and the Ayeyarwady Region to the West. Its population is growing dramatically, with continuous migration of people from other parts of the country, and the city has higher number of registered vehicles than all other major cities [27]. The city is now recognized as the epicenter of the COVID-19 outbreak in Myanmar, because the majority of confirmed cases were detected here [12].

### Selection of specific locations

**Fig. 1:**
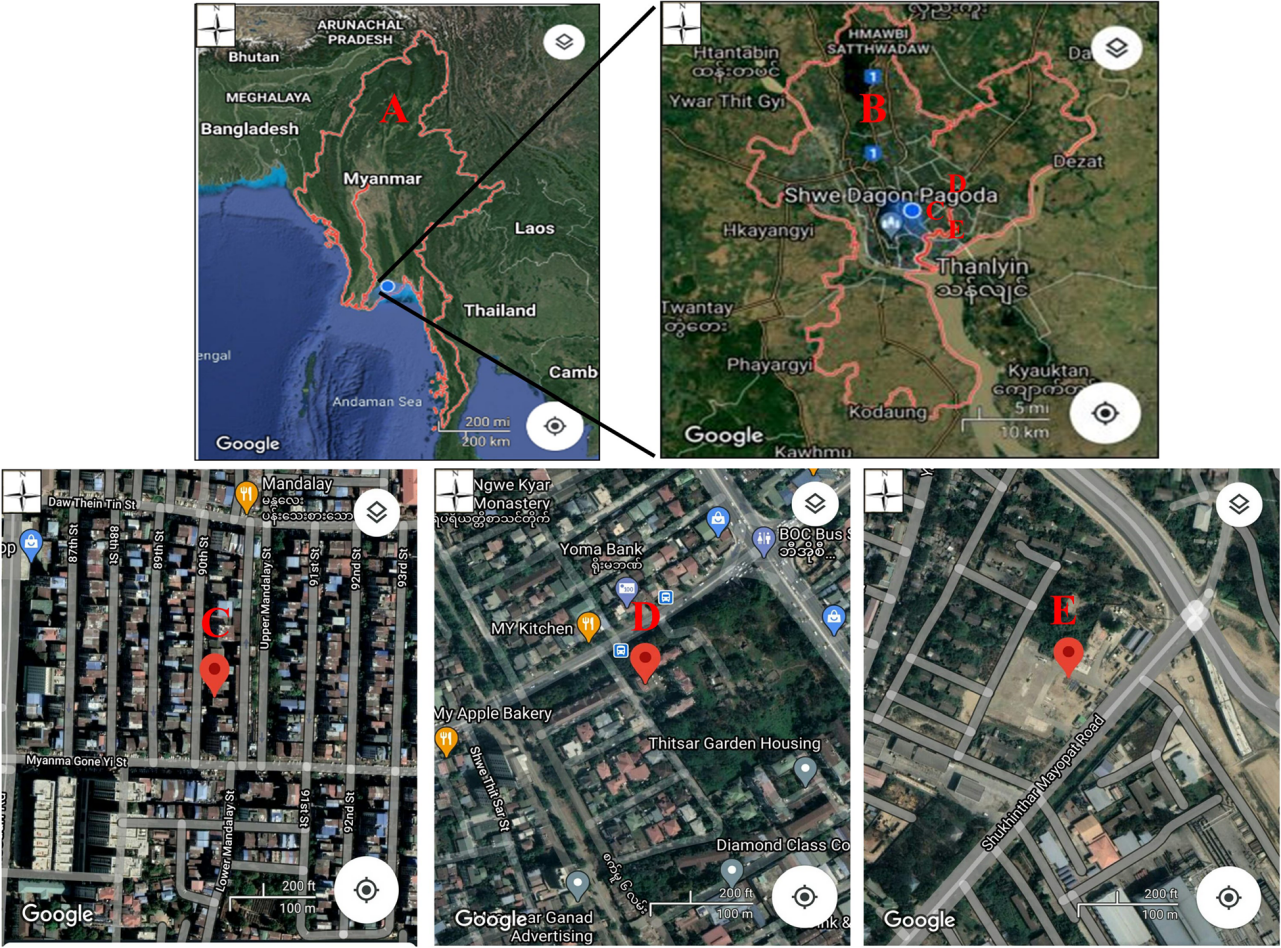
Locations of study sites. **A** Myanmar map, **B** Yangon city map, **C** a crowded semi-commercial area in Mingalar Taungnyunt Township (MGT), **D** a crowded residential area in South Okkalapa Township (SOK), and **E** a quiet residential area in Thaketa Township (TKT)

We randomly selected three study sites with different characteristics; the first, a crowded semi-commercial area in Mingalar Taungnyunt Township (MGT); the second, a crowded residential area in South Okkalapa Township (SOK); and the third, a quiet residential area in Thaketa Township (TKT) (Fig. 1).

The study site in the crowded semi-commercial area (MGT) is surrounded by high-compact buildings in a short lane named “90th street” that connects two busy streets, Myanma Gone Yi Street and Daw Thein Tin Street. Some small businesses, such as computer servicing centers, book shops, and accessories stores, operate in the area. Many shops selling a variety of foods, including tea shops also line each side of the lane, with high local human activity. Moreover, there is usually a high traffic volume around this study site, especially during rush hours. The study site in the crowded residential area (SOK) is close to a busy main road called “Thitsar Road,” on which traffic congestion can be seen frequently. A small private bank, a small restaurant, two food shops, and a bus stop are present in the vicinity of the sensor site. The study site in the quiet residential area (TKT) is located in a small lane, Tarma lane, in a small ward with just a few houses. This quiet residential area is quite away from traffic and public congestion.

### Particulate matter measuring device

**Fig. 2:**
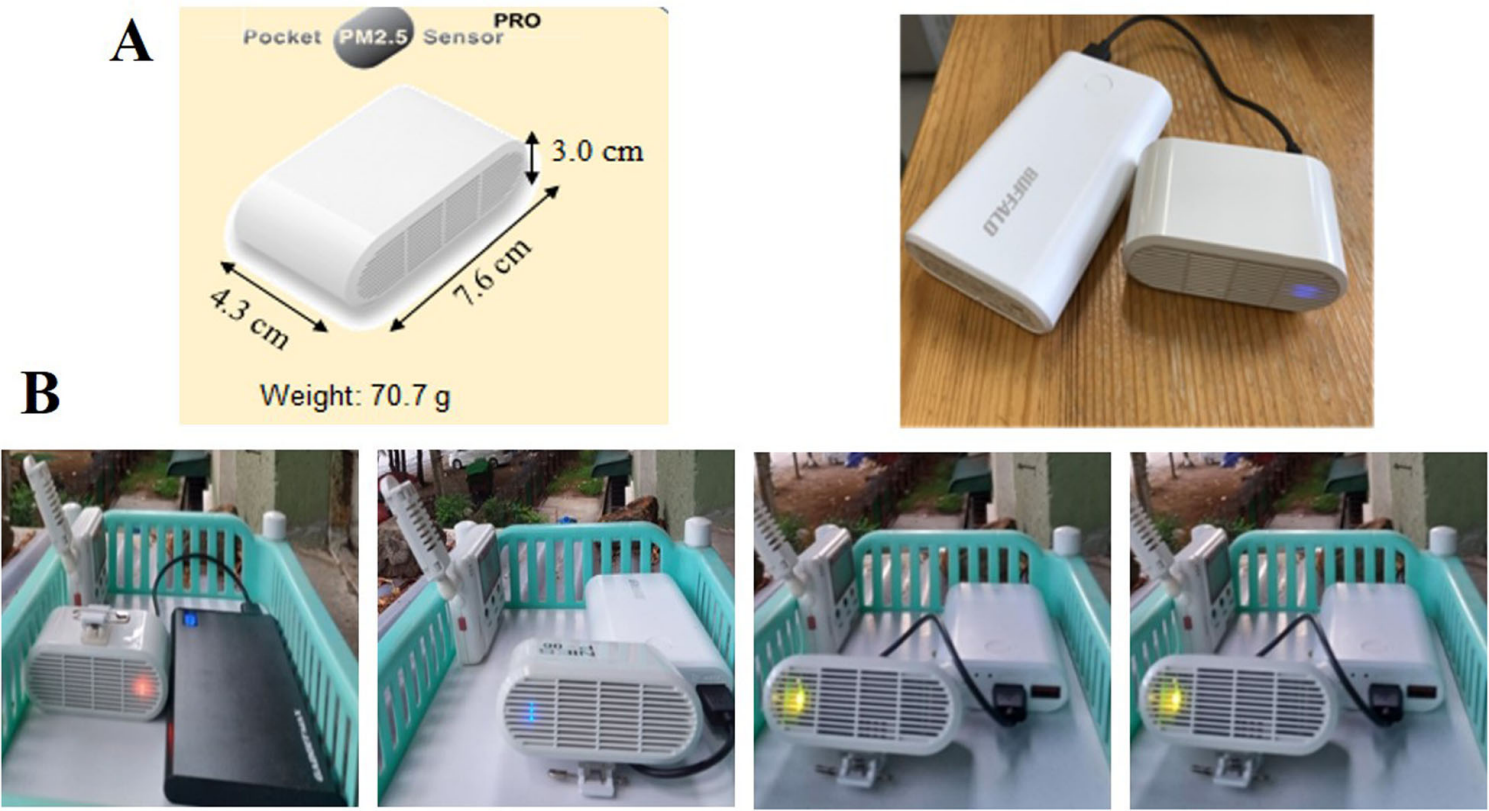
Pocket PM_2.5_ sensor [PRO]. **A** Dimension and color display of the Pocket PM_2.5_ sensor [PRO]. **B** Record of PM concentrations at 8:00 AM on Monday in SOK showing color variations for different levels of PM concentrations. From left to right; first period, second period, third period, and fourth period

The sensor used to measure the ambient PM concentrations, called Pocket PM_2.5_ Sensor [PRO], was designed by the National Institute for Environmental Studies (NIES), Japan, in cooperation with Yaguchi Electric Co., Ltd., (Miyagi, Japan). A total of three Pocket PM_2.5_ Sensor [PRO] were used in this study; one sensor for each study site. The sensor is small, lightweight (70.7 g), and portable (Fig. 2). The detailed specification and validity assessment of the Pocket PM_2.5_ Sensor [PRO] are described in our previous report of a study in which we investigated personal PM_2.5_ exposures among housewives and university female teaching staff [28]. In brief, the Pocket PM_2.5_ Sensor [PRO] is equipped with a global positioning system (GPS) and can be used continuously for 45 h, with a range of measurement from 0 to 999 μg m^−3^. The sensor can measure both PM_2.5_ and PM_10_ every second, and the maximum memory storage is 400 days. The stored data can be downloaded as a comma-separated value (CSV) file. The validity of the Pocket PM_2.5_ Sensor [PRO] was assessed by simultaneously comparing the sensor with PM-712, Kimoto, a fixed real-time monitor [29] set up at the Air Quality Research Station, NIES, Tsukuba, Japan.

### Study period

We measured the ambient PM_2.5_ and PM_10_ concentrations on four occasions for a duration of one week each in the year 2020; namely, February 24 to March 2 (first period), May 10 to May 17 (second period), August 31 to September 6 (third period), and December 7 to December 14 (fourth period). Myanmar, one of the countries of Southeast Asia, has three seasons, namely the summer season (mid-February to mid-May), rainy season (mid-May to mid-October), and winter season (mid-October to mid-February). Therefore, the first and second periods of measurement were at the start and end of the summer season, the third period fell within the rainy season, and the fourth period fell within the winter season.

At each study site, both the Pocket PM_2.5_ Sensor [PRO] (Yaguchi Electric Co., Ltd., Miyagi, Japan) and the data logger (Thermo Recorder, TR-72U, T&D Corp., Nagano, Japan) for measuring the ambient temperature and relative humidity were placed about 3–4 m from the ground. In Yangon, the Stay-At-Home order and other restrictive measures were implemented in the second week of April 2020. The first 1-week measurement period in this study fell before the onset of the COVID-19 pandemic, the second period fell during the first wave of COVID-19, the third period fell within the early period of the major second wave, and the fourth period fell beyond the peak of the second wave. We also downloaded PM data recorded in 2019 and 2020 in an online platform, PurpleAir (https://www2.purpleair.com/), based on measurement using well-calibrated Real-time Air Quality Monitoring laser particle counters. We collected the PM_2.5_ and PM_10_ data for the same periods in 2019 and 2020 uploaded by the PurpleAir sensor installed in the campus of the Myanmar Center for Responsible Business, Ahlone Township, Yangon. Unfortunately, we were unable to use the data before 2019 because our reference PurpleAir sensor was set up and operated only in February, 2019.

### Statistical analysis

The Statistical Package for the Social Sciences (SPSS) version 26 (IBM Corp., Armonk, NY, USA) was used for data entry and statistical analysis. Data cleaning and summarization were effected by checking the descriptive statistics, including the mean, standard deviation (SD), minimum, maximum, medians, interquartile ranges (IQR), histograms, and box plots. The statistical significance level was set at *P* < 0.05. Since both the PM_2.5_ and PM_10_ data for the four periods showed a positively skewed distribution, the data are described with box and whisker plots. We used the non-parametric test, Wilcoxon’s signed-rank test, to compare the ambient PM concentrations among the four periods in each study site. Pearson’s correlation was used to access any linear correlation among the PM data from the PurpleAir sensor and the data from the Pocket PM_2.5_ sensor [PRO], and also used for correlation analysis between the hourly PM concentrations and hourly values of ambient temperature and relative humidity.

## Results

The average values of ambient temperature and relative humidity recorded during the four periods at the three study sites are shown in Table 1. The relative humidity and ambient temperature differed among the study periods. Our correlation analysis between the PM concentrations and the temperature (*r* = 0.11, *P* = 0.003) and relative humidity (*r* = − 0.06, *P* = 0.001) revealed only weak correlations (*r* < 0.25) during all the four study periods at all the three study sites, as well as during the corresponding periods of the previous year (*r* = 0.19, *P* = 0.004 between PM concentration and temperature; *r* = − 0.1, *P* = 0.001 between PM concentration and relative humidity).

**Table 1:** Average values of ambient temperature (T) and relative humidity (RH) in the four measurement periods at the three study sites (2020) and from PurpleAir (2019)

Study site	First period		Second period		Third period		Fourth period	
	T (°C)	RH (%)	T (°C)	RH (%)	T (°C)	RH (%)	T (°C)	RH (%)
MGT	28.1	50.1	28.2	72.0	30.6	74.7	27.1	46.2
SOK	28.8	41.3	32.7	59.8	29.2	79.2	26.1	60.5
TKT	29.7	37.8	30.5	64.8	30.2	73.8	28.6	58.1
PurpleAir (2019)	33.4	41.9	36.1	47.1	31.6	60.7	28.7	40.1

MGT denotes a crowded semi-commercial crowded area; SOK denotes a crowded residential area, and TKT denotes a quiet residential area. First period: February 24 to March 2, at the start of the summer season; second period: May 10 to May 17, at the end of the summer season; third period: August 31 to September 6, during the rainy season; fourth period: December 7 to December 14, during the winter season. The first period was pre-COVID-19, and the second, third, and fourth periods were when COVID-19-restrictive measures were in effect

### Concentrations of particulate matter

**Fig. 3:**
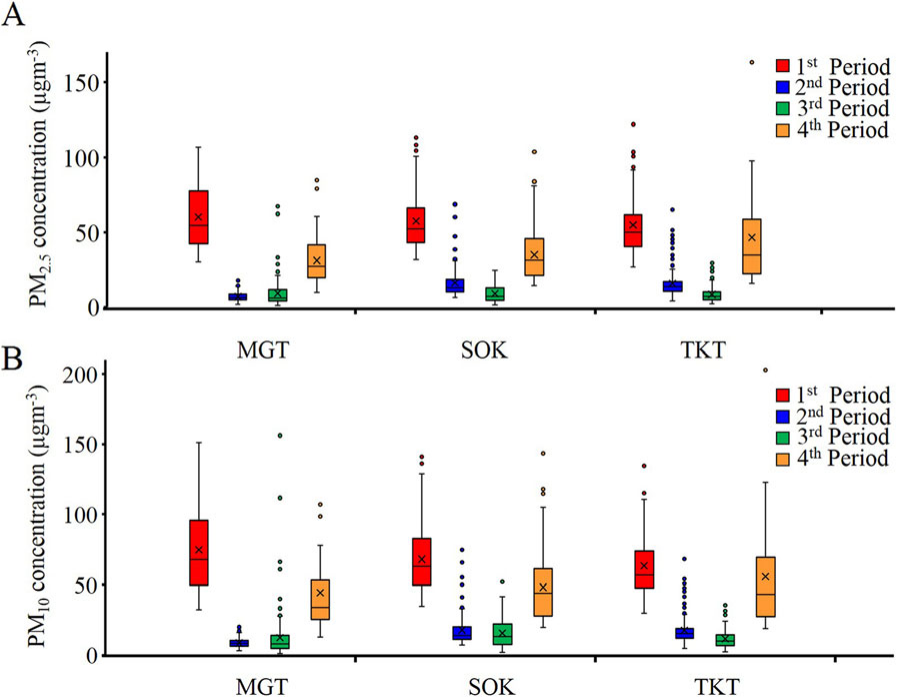
Ambient PM concentrations among four periods of three study sites. **A** PM_2.5_ concentrations and **B** PM_10_ concentrations. Line within the box: median, cross within the box: mean, box: first and third quartile, whiskers: non-outlier range, dots: outliers

Comparisons of the ambient PM_2.5_ and PM_10_ concentrations in the four measurement periods at the three locations are shown in Figs. 3A and B, respectively. At MGT, in comparison to the ambient PM_2.5_ concentration during the first period, the concentrations in the second, third, and fourth periods were significantly lower (*P* < 0.001). In the crowded residential area (SOK) also, the values recorded in the second, third, and fourth periods were significantly lower (*P* < 0.001) than the value recorded in the first period. Similarly, in the quiet residential area (TKT) too, the concentrations recorded in the second, third, and fourth periods were significantly lower (*P* < 0.001) than the concentration recorded in the first period. Similarly, when the PM_10_ concentrations were compared with the value recorded in the first period, significant reductions (*P* < 0.0001) were noted in all the three locations during the three lockdown periods (Fig. 3B). Thus, significant decreases of both ambient PM_2.5_ and PM_10_ concentrations were observed when the COVID-19-restrictive measures were in force as compared to the values recorded prior to the outbreak of the disease, irrespective of the study location.

We also determined the trends in the changes of the PM concentrations during the four periods at the three study sites. For all three study sites, a dramatic reduction of the PM_2.5_ concentration was observed in the second period followed by an even more pronounced drop in the third period. In the fourth period also, there was a substantial reduction of the PM_2.5_ concentration as compared to the value recorded in first period, but the degree of this reduction was not as high as that in the second and third periods. Similar trends were noted for the PM_10_ concentrations at two of the study sites, SOK and TKT, whereas the lowest concentration was found in the second period at MGT (Fig. 3).

### Percent changes in particulate matter concentrations

We performed further analysis to determine the relative percent changes (%) of the PM concentrations during the three periods of measurement after the onset of the COVID-19 pandemic, using either the measured values in the first period just prior to the COVID-19 outbreak or the measured values during the same periods in 2019 as reference. The percent changes were determined (1) by expressing the concentration difference between values measured in the first period and those measured in the second, third, or fourth periods as a fraction of the value recorded in the first period and multiplying by 100, and (2) by expressing the concentration differences between the second, third, and fourth periods during the COVID-19 pandemic and the corresponding periods in 2019 as a fraction of the values measured in the corresponding periods in 2019 and multiplying by 100.

As mentioned earlier in the “Materials and Methods” section, we also collected data on the PM concentrations from the online platform, PurpleAir. There was a strong linear correlation between the PM data recorded in PurpleAir (2020) and the data obtained from Pocket PM_2.5_ sensor [PRO] set at each study site; *r* = 0.96 at MGT, 0.99 at SOK, and 0.93 at TKT, suggesting that the recorded data using different types of sensors are comparable. Table 2 shows the percent changes in the PM concentrations in the second, third, and fourth periods relative to the values recorded during the same periods in 2019. This comparison revealed marked percent reductions in both PM_2.5_ and PM_10_ concentrations during the second period alone. A minimal reduction or even a slight percent increase was seen during the third period and a moderate reduction during the fourth period.

**Table 2:** Percent changes in the ambient particulate matter concentrations relative to the values measured during the same periods in 2019

Study sites	Percent changes in PM concentration (%)		
	Second period	Third period	Fourth period
PM2.5			
MGT	− 75.4	− 18.3	− 37.7
SOK	− 60.3	− 19.1	− 33.3
TKT	− 62.0	− 23.1	− 11.3
PM10			
MGT	− 83.4	+ 0.5	− 30.9
SOK	− 64.3	+ 26.6	− 25.3
TKT	− 65.4	− 6.5	− 13.4

(−) Percent reduction; (+) percent increase

**Fig. 4:**
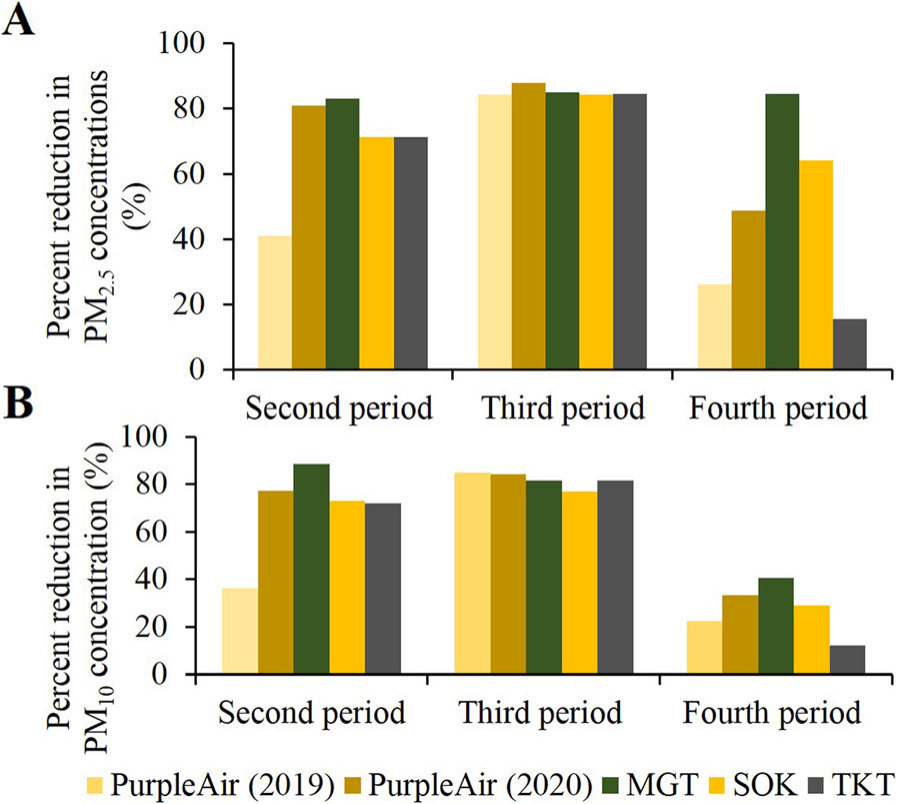
Comparison of the percent reductions among the five data sources. **A** PM_2.5_ concentration and **B** PM_10_ concentration. The five data sources are PurpleAir (2019), PurpleAir (2020), MGT, SOK, and TKT. Using the period, February 24 to March 2 (first period) as reference, the percent reductions for the other three study periods, i.e., May 10 to May 17, August 31 to September 6, and December 7 to December 14, were calculated

Figure 4 shows a comparison of the percent changes in the latter three periods, that is, May 10 to May 17, August 31 to September 6, and December 7 to December 14, for the five data sources, namely PurpleAir (2019), PurpleAir (2020), MGT, SOK, and TKT, using the values measured in the first period (February 24 to March 2) as reference. In 2020, during the pandemic, a significant percent reduction (above 70%) in both PM_2.5_ and PM_10_ concentrations were observed at all the three study sites in the second and third periods. However, in the fourth period, the percent reductions of the PM concentrations were lower as compared to those in the preceding two periods. When compared to the data from PurpleAir (2019), the percent reductions in the second and fourth periods were lower than the data recorded in PurpleAir (2020), MGT, SOK, and TKT. However, in the third period, the percent reductions were almost equal (round about 80%) among the five data sources, i.e., the percent reductions in 2019 were similar to those in 2020.

### Daily mean concentrations of particulate matter

**Fig. 5:**
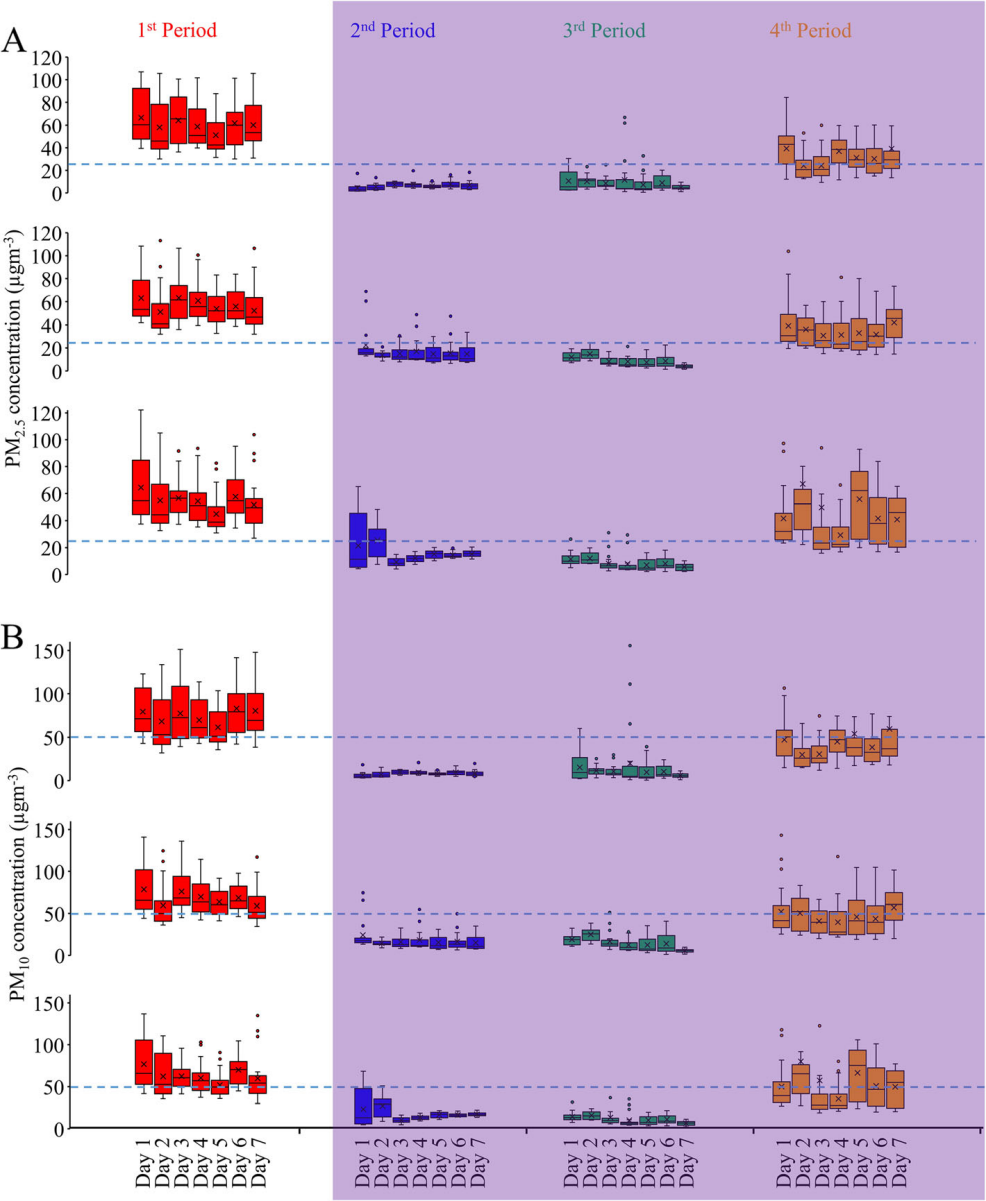
Mean daily PM concentrations during the four periods at the three study sites. **A** Mean daily PM_2.5_ concentration in MGT (upper channel), SOK (middle channel), and TKT (lower channel). **B** Mean daily PM_10_ concentration in MGT (upper channel), SOK (middle channel), and TKT (lower channel)

Figure 5 shows the variations in the 24-h average concentrations of PM_2.5_ and PM_10_ in the four periods at the three sampling sites. At all three sites, the daily mean PM_2.5_ and PM_10_ concentrations in the first period, i.e., before the COVID-19 outbreak/lockdown, were invariably above the WHO-recommended limits (25 μg m^−3^ for PM_2.5_ and 50 μg m^−3^ for PM_10_) [3]. However, the daily mean PM_2.5_ and PM_10_ concentrations were consistently below the set values on almost all the seven days of both second and third periods at all the three study sites. In the fourth period, on most of the days, the PM_10_ concentrations were below the set limits, while daily mean PM_2.5_ concentrations were above the set limits.

## Discussion

During the first period of measurement, prior to the implementation of the COVID-19-restrictive measures, the recorded PM data at the three study sites were in accordance with our classification; the mean PM_2.5_ and PM_10_ concentrations were the highest in MGT, which was the most crowded area in terms of both vehicular and pedestrian traffic, while they were the lowest in TKT, the least crowded of the test areas (Fig. 3). However, this ordering of PM concentrations in the three areas was lost during the period of implementation of the restrictive measures, and even reversed in MGT, the site at which the lowest PM concentrations among the three sites were recorded during the second and fourth periods of measurement. Some crowded places or densely polluted metropolitan cities with higher anthropogenic emissions of PM due to high traffic volumes and crowded local human activities showed a greater degree of reduction of PM concentrations when COVID-19-restrictive measures were in force than less urbanized cities or quiet places [30, 31].

The finding of a significant decrease in PM concentrations during the period in which COVID-19-restrictive measures were in effect is consistent with many recent reports worldwide [13–17] and restricted vehicular traffic movement and temporary closure of universities, restaurants, food shops, factories, and industries are common possible explanations for such a finding. On the contrary, only a slight reduction or even an unexpected increase in PM concentrations during lockdown periods has also been reported [18–21]. These discrepant results could be due to differences in the types of restrictive measures implemented, such as total lockdown, community lockdown, large-scale social distancing and movement control order, differences in the enforcement periods, local meteorological conditions, and the intensity of pre-existing anthropogenic emissions at the respective study sites.

In our study, a noticeable percent reduction (above 70%) was observed at the study sites during the second and third periods of measurement, as compared to the fourth period. Notably, in MGT, a crowded semi-commercial area showed a consistent degree of reduction in PM_2.5_ in the second period (82.9%) and third period (84.8%) of measurement, which could have been a result of closure of almost all shops and stores at these sites and prohibition of private vehicular movement on the streets, except for those of house owners in the area during these periods. We observed inconsistent changes of the PM concentrations among the three measurement periods during which COVID-19-restrictive measures were in place, and this observation was in line with some previous reports [18, 22].

While some researchers compared the PM data measured during the lockdown periods with data obtained during a period preceding the lockdown in 2020 [32], many investigators compared the PM data obtained while COVID-19-restrictive measures were in effect with those measured at the same time in the previous one or more years [18, 33, 34], and the comparisons were done in terms of percent changes of PM concentrations. It is conceivable that the variations in meteorological parameters during the same period over different years would be relatively less pronounced than the variations between the periods before and during the lockdown within the same year [33].

In Yangon, Stay-At-Home order, Work-From-Home order, and other restrictive measures, including closure of universities, factories, and restaurants allowing take-away service only, were implemented from the second week of April 2020. According to the Road Transportation Administration Department (RTAD), in 2020, there were about 540,000 registered vehicles in Yangon, among which 350,000 automobiles, that is, over two-thirds, were private cars and about 41,000 vehicles were public transport vehicles [27]. Because of the Stay-At-Home and Work-From-Home orders, there could have been a dramatic reduction in the number of both private and public transport vehicles on the road, resulting in a recognizable reduction of the traffic volume.

The percent change in PM concentrations in the second period, that is, May 10 to May 17, were consistently found to be greatly reduced, regardless of the type of comparison (Table 2 and Fig. 4). This period in May is a period of transition from the summer to the rainy season and the percent changes in the PM concentrations were still high even when the comparison was made with the data obtained in the corresponding period of 2019, with similar meteorological conditions. Therefore, such an obvious degree of reduction in this second period of measurement could be directly attributable to the strict obligation of the citizens to conform to the restrictive measures during first wave of COVID-19, resulting in a dramatic reduction in the anthropogenic emissions of PM.

During the third period of measurement, that is, August 31 to September 6, as described earlier, the extent of reduction of the absolute PM concentrations was even more pronounced (Fig. 3). Moreover, the percent reduction was also high when the comparison was made using the data obtained during the first period, before the onset of the COVID-19 pandemic, as reference (Fig. 4). However, the percent changes in this period relative to the data obtained during the corresponding period of 2019 showed only minimal reduction or even a percent increase (Table 2). Another interesting finding was that when the percent reductions among the five data sources, namely PurpleAir (2019), PurpleAir (2020), MGT, SOK, and TKT, were compared, the values were almost equal for both PM_10_ and PM_2.5_ concentrations (Fig. 4). This finding suggests that in 2019, even in the absence of COVID-19-restrictive measures, an obvious percent reduction occurred during the period from August 31 to September 6. In fact, the third period fell during the rainy season in Yangon, when precipitation of PM by rain could occur. Consequently, in the third period of measurement, when COVID-19-restrictive measures were in force, seasonal influence could have been the predominant factor contributing to the reduction in ambient PM pollution, although reduced anthropogenic emissions due to the restrictive measures could also have contributed to the reduction.

The fourth period of measurement, namely December 7 to December 14, fell within the winter season. In contrast to the case during the rainy season, the temperature inversion phenomenon, a favorable condition for ambient PM concentrations, commonly occurs during the winter season. Moreover, burning of dry leaves during the winter season could also be a possible additional source of PM. Such seasonal factors could have been responsible for the lower percent reductions in the fourth period as compared to the two preceding periods of measurement (Fig. 4). However, the percent reductions could not be greatly determined by seasonal pollutant dispersion, because unlike in the third period, there was an inconsistency in the percent reductions in the fourth period; the percent reductions of PM_2.5_ and PM_10_ in TKT were lower than those in PurpleAir (2019), whereas those in the remaining data sources were greater (Table 2 and Fig. 4). A renovation project at the Thanlyin Bridge, about 7–8 km away from the TKT site, resumed in November and this emission source could be a possible reason for the highest PM concentration and lowest percent reduction in the quiet residential quiet area. During the second wave of COVID-19, although the COVID-19-restrictive measures were enforced again, many factories and construction sites, restaurants, and food shops resumed their business after being cleared to do so according to the guidelines set by the MOHS. Although prohibition of mass gatherings was still in effect, there was a resurgence of human activities. Therefore, the decline in the percent reductions in the fourth period could be attributable, at least in part, to some relaxation of the restrictive measures, with a lower degree of compliance with orders by the citizens and in part, by the presence of weather conditions that favor PM dispersion.

Some studies have also taken into consideration seasonal variations while describing the changes in PM concentrations during the COVID-19 pandemic. In the study reported by Hashim et al. (2020), the investigators compared the average PM concentrations in Baghdad, Iraq, during five periods; the first period before the enforcement of a lockdown, and the remaining four periods during partial or total lockdown. They observed that the PM_2.5_ and PM_10_ concentrations were the lowest during the first partial and total lockdowns among the five periods. They speculated the following possible reasons for this finding; the citizens’ compliance with the lockdown measures during that first lockdown period contributed to the large decline of PM concentrations during that period, and the dry hot climate during the summer resulted in the relative increase of PM concentrations in the subsequent lockdown periods [18]. In a report from Thailand [32], the ambient PM concentrations were compared among three measurement periods; pre COVID-19, early COVID-19, and while a work-from-home order was in place. An unexpected increase in the ambient PM concentrations was noted in the early COVID-19 period, during which only personal hygiene measures were encouraged, without other strict restrictive measures, and there was also the seasonal transition from winter to summer.

Meteorological factors, such as the ambient temperature, relative humidity, wind speeds, precipitation, radiation, and ambient pressure could also exert an influence on the ambient PM concentrations [35]. In our study, the ambient temperature and relative humidity were measured and their correlations with the PM concentrations were evaluated. In spite of revealing significant level of *P* value, only weak correlations (*r* < 0.25) were noted during all the four periods of three study sites (*r* = 0.11, *P* = 0.003 between PM concentration and temperature; *r* = − 0.06, *P* = 0.001 between PM concentration and relative humidity). A previous study also showed that during the COVID-19 pandemic, these two meteorological parameters were only weakly correlated with the PM concentrations (*r* < 0.25), indicating that they contributed little to the ambient PM concentrations [36]. Although we found an obvious difference in the temperature and relative humidity between the COVID-19 pandemic year (2020) and the previous year (2019) (Table 1), the aforementioned correlations were also weak for 2019 data (*r* = 0.19, *P* = 0.004 between PM concentration and temperature; *r* = − 0.1, *P* = 0.001 between PM concentration and relative humidity). These findings could be attributable to the contributions of other meteorological factors rather than the two aforementioned variables to the ambient PM concentrations.

**Fig. 6:**
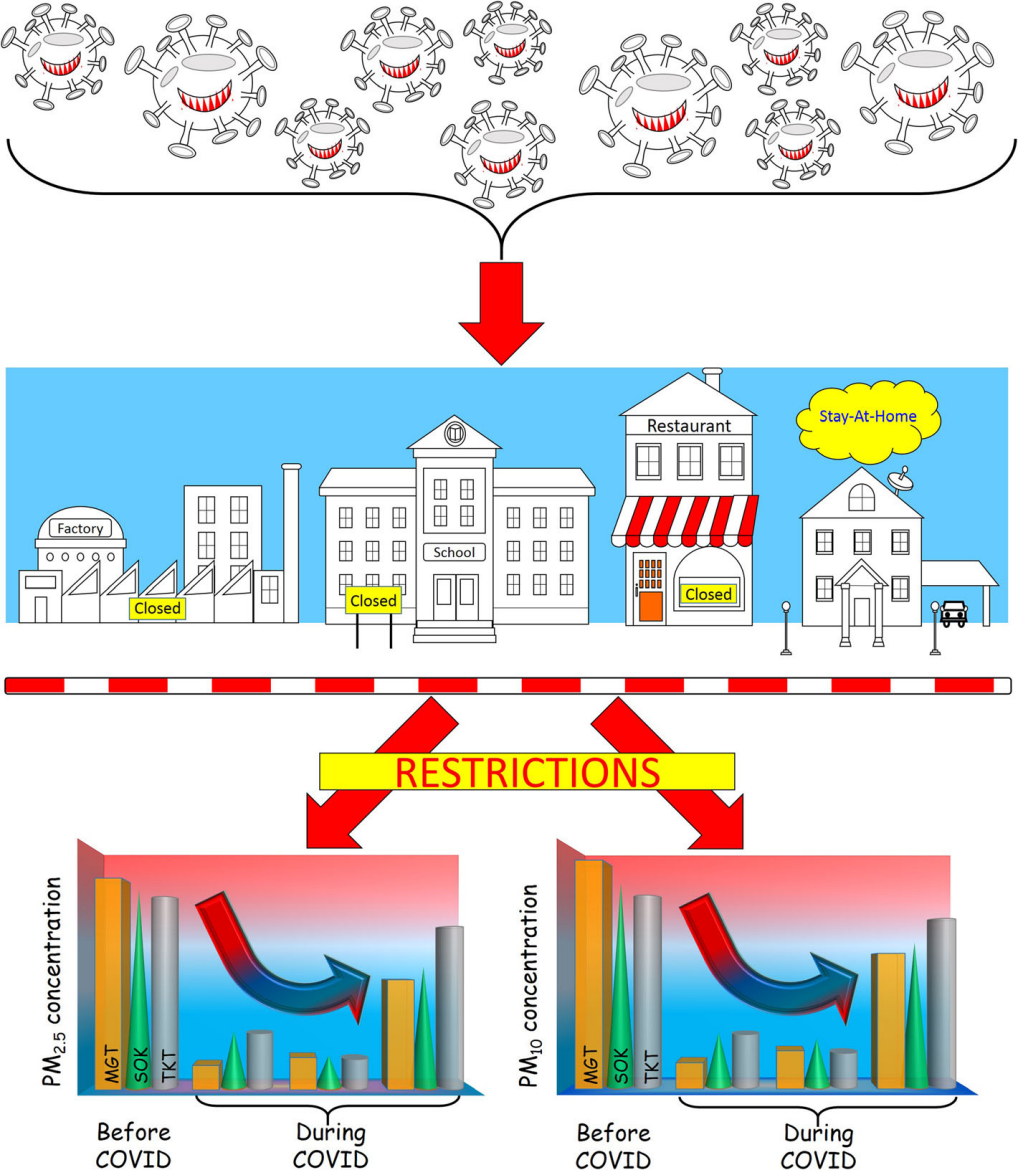
Summarized results. Summarization showing the effect of COVID-19-restrictive measures on ambient particulate matter pollution in Yangon, Myanmar

Yangon city indeed suffers from PM-related air pollution. During the period from January 25 to January 29, 2018, we assessed the ambient PM_2.5_ and PM_10_ concentrations in seven townships of the city, and found that the mean PM concentrations were over the WHO guideline limits [23, 24]. The average annual PM_2.5_ concentration (weighted by the population) in the city in 2019 was 31 μg m^−3^, exceeding the annual mean PM_2.5_ exposure threshold of 10 μg m^−3^ set by the WHO. In regard to the ranking of regional capital cities of the world according to the PM_2.5_ exposure level, Yangon is placed 19th out of 85 capital cities across the world, and ranks third among cities in the Southeast Asia region [4]. In our study, the daily mean PM_2.5_ and PM_10_ concentrations measured in the period prior to the onset of the COVID-19 pandemic were consistently over the WHO-recommended limits (Fig. 5). Conversely, the mean daily PM_2.5_ and PM_10_ concentrations were below the set limits on almost all seven days of both the second and third periods of measurement, when COVID-19-restrictive measures were in effect. Therefore, in contrast to the previous years, Yangon city experienced a profound improvement of PM-related air quality in 2020, and this appears to be, in all probability, due to the restrictive measures proposed for COVID-19 containment. Moreover, as compared to previous reports from studies conducted worldwide, the percent reductions in our study were relatively higher. This could be due to the fact that the city, in which no prior proactive air pollution control measures were in place, experienced a rapid and effective restriction of anthropogenic emissions for a very first time during the period of enforcement of COVID-19-restrictive measures (Fig. 6).

The United States Air Quality Index (USAQI) is the most widely used for assessment of the ambient air quality. The average 24-h PM_2.5_ concentrations are converted into six categories of AQI, where higher values indicate a higher health risk [4]. In our study, the air quality during the first period of measurement prior to the onset of COVID-19 fell into either the unhealthy category (55.5–150.4 μg m^−3^) or the category of “unhealthy for sensitive group (USG),” such as children, elderly persons, and patients with cardiovascular disease (35.5–55.4 μg m^−3^). However, improvement in air quality became apparent during the second and third periods of measurement, when COVID-19 lockdown was in place, and the AQI category became moderate (12.2–35.4 μg m^−3^) or even good (0–12.1 μg m^−3^), i.e., the air quality became satisfactory and posed little or no risk. Unfortunately, on a few days during the fourth period, the AQI was categorized as USG.

In addition, as fine particles are more harmful than coarse particles, higher PM_2.5_/PM_10_ ratios may result in serious air pollution, whereas the lesser the ratio, the lesser the possibility of poor air quality [30]. A higher ratio implies predominant contribution of PM_2.5_, which is generally ascribed to primary pollution by anthropogenic emissions, while a lower ratio suggests a greater contribution of coarse particles, which mainly arises from natural sources [37]. In a study from South Korea, after the implementation of social distancing, the PM_2.5_/PM_10_ ratio decreased from 0.66 to 0.4 in Seoul and 0.68 to 0.54 in Daegu city, and this finding was explained by a decrease in anthropogenic emissions [17]. In our study also, the PM_2.5_/PM_10_ ratios declined during the COVID-19 measurement periods as compared to the pre-COVID-19 values; from 0.82 to 0.79 in MGT, from 0.86 to 0.82 in SOK, and from 0.88 to 0.82 in TKT. This finding indicates that when the COVID-19-restrictive measures were in place, the PM-related air quality improved, and more specifically, that reducing human activities favors reduction of the anthropogenic sources of PM_2.5_ rather than PM_10_. A study in Wuhan city showed that not only did the mass concentration of PM_2.5_ reduce, but its chemical composition also altered during the period of enforcement of COVID-19-restrictive measures [38]. Although we only assessed the mass concentrations of PM in this study, we propose to analyze the PM compositions in Yangon cut using a high-volume sampler in the future [39].

## Conclusions

We took advantage of the rare opportunity, during the COVID-19 pandemic, to investigate the atmospheric PM response to rapid, widespread anthropogenic emission reductions. Our results revealed a remarkable reduction in both the PM_2.5_ and PM_10_ concentrations while COVID-19-restrictive measures were in force in Yangon city, indicating that these restrictive measures had a positive impact on the ambient PM concentrations. The changes in the PM concentrations were considered to be largely attributable to the reduction in anthropogenic emissions as a result of the restrictive measures in place, but there could also have been seasonal influences. The scenario of reduction of PM concentrations while restrictive measures were in force during the COVID-19 pandemic highlights the fact that the compliance of citizens with the implementation of environmental policies on air quality could be very essential for effective reduction of anthropogenic emissions contributing to pollution. Therefore, when devising an action plan for limiting PM emissions, it is necessary to first raise the awareness of the public about the health risks associated with air pollution. According to our findings, the restrictive measures, including the Stay-At-Home order, were brilliant examples of ways to control the sources of emission. Thus, frequent implementation of a weekly or biweekly or Telework policy may be a feasible way of reducing PM pollution and further longitudinal studies to determine the effects of such short-term application of restrictive measures on the incidence of PM-related health problems is also recommended.

## Data Availability

The datasets are not opened to the public but are available from the corresponding author on reasonable request.

## Abbreviations

PM: Particulate matter
COVID-19: Coronavirus disease-2019
NIES: National Institute for Environmental Studies
SARS-CoV-2: Severe acute respiratory syndrome coronavirus 2
WHO: World Health Organization
MOHS: Ministry of Health and Sports
MGT: Mingalar Taungnyunt
SOK: South Okkalapa
TKT: Thaketa
GPS: Global positioning system
CSV: Comma-separated value
SPSS: Statistical Package for the Social Sciences
SD: Standard deviation
IQR: Interquartile range
T: Temperature
RH: Relative humidity
USAQI: United State Air Quality Index
AQI: Air Quality Index
USG: Unhealthy for sensitive group
